# Sex-specific genetic architecture in response to American and ketogenic diets

**DOI:** 10.1038/s41366-021-00785-7

**Published:** 2021-03-15

**Authors:** Anna C. Salvador, Danny Arends, William T. Barrington, Ahmed M. Elsaadi, Gudrun A. Brockmann, David W. Threadgill

**Affiliations:** 1 Department of Molecular and Cellular Medicine, Texas A&M Health Science Center, College Station, TX USA; 2 Department of Nutrition, Texas A&M University, College Station, TX USA; 3 Züchtungsbiologie und molekulare Genetik, Albrecht Daniel Thaer-Institut, Berlin, Germany; 4 Department of Biochemistry & Biophysics, Texas A&M University, College Station, TX USA

**Keywords:** Genetics, Nutrition disorders

## Abstract

**Background/objectives:**

There is a growing appreciation for individual responses to diet. In a previous study, mouse strain-specific responses to American and ketogenic diets were observed. In this study, we searched for genetic variants underlying differences in the responses to American and ketogenic diets between C57BL/6J (B6) and FVB/NJ (FVB) mouse strains.

**Results:**

Genetic mapping of fat and lean mass gain revealed QTLs on Chromosome (Chr) 1 at 191.6 Mb (*Fmgq1*) (*P* < 0.001, CI = 180.2–194.4 Mb), Chr5 at 73.7 Mb (*Fmgq2*, *Lmgq1)* (*P* < 0.001, CI = 66.1–76.6 Mb), and Chr7 at 40.5 Mb (*Fmgq3*) (*P* < 0.01, CI = 36.6–44.5 Mb). Analysis of serum HDL cholesterol concentration identified a significant (*P* < 0.001, CI = 160.6–176.1 Mb) QTL on Chr1 at 168.6 Mb (*Hdlq1*). Causal network inference suggests that HDL cholesterol and fat mass gain are both linked to *Fmgq1*.

**Conclusions:**

Strong sex effects were identified at both *Fmgq2 and Lmgq1*, which are also diet-dependent. Interestingly, *Fmgq2 and Fmgq3* affect fat gain directly, while *Fmgq1* influences fat gain directly and via an intermediate change in serum cholesterol. These results demonstrate how precision nutrition will be advanced through the integration of genetic variation and sex in physiological responses to diets varied in carbohydrate composition.

## Introduction

Efforts to provide individualized dietary recommendations based on genetic markers have been underway for several years. However, these efforts have largely detected alleles with small-effect sizes that are not clinically actionable [1–3]. As with studies investigating responses to other environmental factors, responses to diet are likely not due to the action of single alleles with a large effect, but rather the sum of multiple small-effect alleles, interactions among alleles, epigenetic modifications, and sex.

Recently, our group observed strong mouse strain-specific differences in response to feeding different human-relevant diets [4–6]. This initial study provided evidence for striking differences between C57BL/6J (B6) and FVB/NJ (FVB) mice in response to high-fat diets varying in carbohydrate content. Although studies increasingly consider the role of sex as a biological variable, the role of sex in the response to diets with varied macronutrient contents has been understudied [7].

To further investigate the genetic origin of differential response to carbohydrate restriction and sensitivity to high-fat diets, we generated an intercross population (F2) between B6 and FVB. All F2s were fed either an American (35% of energy from fat, 50% from carbohydrate) or a ketogenic (84% of energy from fat, 0% from carbohydrate) diet and changes to body composition and serum cholesterol concentrations were measured. In this population, we performed genome-wide linkage analysis to elucidate the genetic architecture contributing to differential responses to the specific diets. The data obtained provide evidence for genetic loci that not only directly affect body composition response but also loci that indirectly affect response through differences in serum cholesterol concentration. In addition, significant sex differences in the effects of the identified quantitative trait loci (QTL) were detected.

## Methods

### Animals and diets

Initially, we screened 6-week-old B6 and FVB mice for their response to American (35% of energy from fat, 50% from carbohydrates) and ketogenic (84% of energy from fat, 0% from carbohydrates) diets after a 6-month feeding trial. These strains have previously exhibited significantly different responses to these two diets [4]. Detailed diet compositions are provided in Supplementary Table 1. B6 females were crossed with FVB males to generate F1 mice and subsequently intercrossed to generate the F2 population. Four-week-old F1s and 3–5 week-old F2s were screened for their response to American and ketogenic diets during a 3-month feeding trial.

For the feeding trials, mice were randomly assigned to one of the two diet groups. Half of the B6, FVB, F1, and F2 mice were placed on American diet (B6: 11 males, 9 females, FVB: 10 males, 10 females, F1: 6 males, 6 females, F2: 102 males, 122 females) and a half on the ketogenic diet (B6: 9 males, 10 females, FVB: 10 males, 10 females, F1: 6 females, 9 males, F2: 126 males, 119 females). Researchers were not blinded to diet assignments. All animals were maintained in accordance with Texas A&M University Institution Animal Care and Use Committee guidelines at 22 °C under a 12-h light cycle. At the end of the feeding trial, mice were euthanized by carbon dioxide asphyxiation, blood was collected, and tissues were harvested and immediately flash-frozen in liquid nitrogen.

### Phenotyping

Echo magnetic resonance spectroscopy (MRI) (EchoMRI, Houston, TX, USA) was used to measure the fat and lean mass of all individuals. During the initial screen of the B6 and FVB strains, body weight and body composition were measured at a 3-month time point of the 6-month feeding trial. In the F1 population, body weight and body composition were measured at the beginning and end of the 3-month feeding trial. The fat percentage of total body weight was calculated at the 3-month timepoint for B6, FVB, and F1 populations. Fat percentage is the percentage of total body fat mass measured by MRI relative to body weight at the time of the MRI measurement. In the F2 population, body weight and body composition from before and after the 3-month feeding trial allowed for changes in fat and lean mass to be calculated. Fat and lean mass gains were calculated as the difference in fat and lean mass prior to the feeding trial and after 3 months on the assigned diet.

In the F2 population, total cholesterol, HDL, and LDL fractions, as well as APOA2 were measured in serum obtained from blood at sacrifice at the end of the feeding trial. Total cholesterol, HDL, and LDL measurements were performed in duplicate using the EnzyChrom AF HDL and LDL/VLDL Assay kit (BioAssay Systems, Hayward, CA, USA). APOA2 measurements were performed in duplicate in a subset of the F2 population with the highest and lowest serum HDL cholesterol concentration (11 males, 27 females), using the Mouse Apolipoprotein A2 ELISA kit (ABclonal cat no. RK02605, Woburn, MA, USA).

### Genotyping

The F2 population was genotyped on the Mouse Universal Genotyping Array (MUGA) that includes 7854 SNP markers [8]. Markers that were not polymorphic between B6 and FVB were removed from the dataset and uncertain genotype calls for individuals (GenCall score quality metric <0.7) were set to missing. The remaining markers were used to generate a genetic map to check for problematic markers and/or sample DNAs. After all corrections, 1667 markers were used for the association analyses. Updated MUGA marker annotation was obtained from Dr. Karl Broman (https://kbroman.org/MUGAarrays/new_annotations.html).

### Heritability calculations

Broad-sense heritability for body fat percentage was calculated as the ratio of total genetic variance (variance in the F2 population) to environmental variance (variance in the F1 population).

### Quantitative trait loci analysis

Outliers can have a strong influence on the results of QTL analyses. We observed biologically implausible errors in data that were greater than three standard deviations from the mean and suspect that these reflect the technical errors in the measurements. As such, outliers were defined as individuals with phenotypes that were more than three standard deviations away from the mean for each sex and phenotype in the F2 population. Outliers were set to missing. This procedure was repeated iteratively to discover all outliers in the data. Pearson’s correlation between phenotypes was determined after correcting for sex and diet effects (Supplementary Fig. 1). Code available by request.

The combined model includes all F2s, both sexes, and both diets (y~ sex * diet + marker). QTL peaks with a logarithm of the odds (LOD) greater than thresholds determined by 10,000 permutations were considered genome-wide significant (*P* < 0.05, LOD > 3.90) or highly significant (*P* < 0.01, LOD > 4.70). A LOD drop of 1.5 LOD from the top marker was used to determine the 95% confidence intervals, or support intervals, for each QTL. Linear models using ANOVA was used to check for any interactions between sex and/or diet with the top markers of each QTL. The variance explained by the top markers at each QTL was calculated by dividing the sum of squares of the model including the top marker by the total sum of squares of the model without QTL.

### Candidate gene analysis using KEGG

KEGG pathways are a collection of pathway maps that reflect known genetic and metabolic relationships. All genes within each significant QTL confidence interval were annotated with KEGG pathway identifiers. We further characterized our candidate genes by KEGG pathways related to glucose, insulin, fatty acids, adipocytes, cholesterol, obesity, diabetes mellitus, metabolic syndrome, and digestion and absorption of carbohydrates, fats, and proteins. A comprehensive list of KEGG pathway queries is provided in Supplementary Table S4.

### Conditioned and unconditioned linkage analysis

We performed a set of conditional QTL scans using traits as covariates in the analysis of other traits based on the individual QTL that were identified for each trait and suspected biological relationships. If conditioning the genome scan with a covariate resulted in a significant increase or decrease in the absolute value of the LOD score, it was interpreted that the traits are causally related to one another. When comparing conditioned and unconditioned QTL scans, an increase or decrease in the LOD of at least 2.0 corresponds to a 5% type I error rate [9, 10]. Only conditioned genome scans that resulted in a change of 2.0 LOD were considered pleiotropic, or shared, QTL between the two traits.

### Causal network inferences

For traits with overlapping QTL we made causal inferences in networks based on methods described elsewhere [11]. Briefly, the first trait (T1) was regressed on the second trait (T2) and T2 was regressed on T1 in order to obtain the residual of each trait after adjusting for the other (R1 and R2). A bivariate *t* test between R1, R2, and the shared locus was used to infer the causal network among them. *P* values were Holm–Bonferroni corrected for the number of tests (i.e., number of residuals tested = 2) where *P* = α_0.05_/(number of tests – rank of *i*th hypothesis +1). A *P* value < 0.05 is considered significant. An initial pathway describing the relationship between T1 and T2 was defined based on the inferred causal networks, and the predicted causal models were compared with the Akaike’s information criterion (AIC) and Bayesian information criterion (BIC). A lower AIC and BIC indicates a better model.

### Structural equation modeling

Structural equation modeling was used to illustrate observed and unobserved relationships between the QTL models. The models were refined until all path coefficients were significantly different from zero. Linear models using ANOVA was used to check for the amount of variation explained by each predictor in the structural model. The proportion of variation explained with the predictors modeled was calculated by dividing the sum of squares of the model including the predictor by the total sum of squares without the predictor.

### Quantitative PCR (qPCR)

RNA from flash-frozen gonadal fat and liver were extracted using the simplyRNA Tissue kit on a Maxwell AS3000 (Promega). cDNA was generated using the Transcriptor First Strand cDNA Synthesis kit (Roche). qPCRs were performed on a LightCycler 480 (Roche) and CFX384 (BioRad) with LightCycler 480 SYBR Green I Master reagent (Roche). *B2m* was used as a housekeeping gene to correct for starting amounts of cDNA in both gonadal fat and liver. Primers were purchased from Integrated DNA Technologies as custom DNA oligos and sequences are provided in Supplementary Table S2.

## Results

### Genetic background and sex modulate parental strain fat differences

**Fig. 1:**
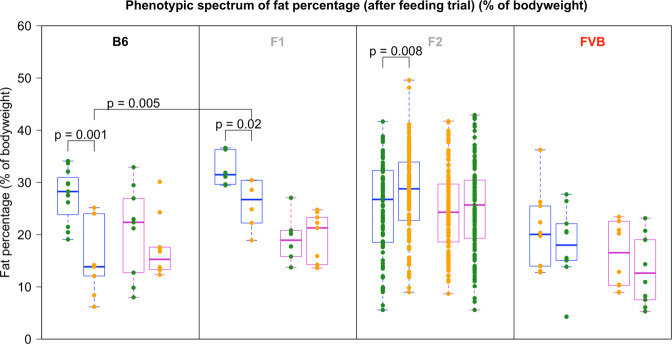
Fat percentage (after feeding trial) (% of body weight). Orange dots indicate a ketogenic diet. Green dots indicate American diet. Blue (male) and pink (female) boxes denote sex. B6 males on the ketogenic diet have a lower percentage of body fat than B6 males on the American diet (Welch’s two-sample *t* test when variances are unequal). This trend persists in the F1 population where F1 males on the ketogenic diet have a lower percentage of body fat than F1 males on the American diet. The F1 males on the ketogenic diet also have a significantly higher percentage of body fat than B6 males on the ketogenic diet.

The fat percentage of B6 males consuming an American diet was 1.77-fold higher compared to B6 males consuming a ketogenic diet (B6 male American: 27.4 ± 5.2%, B6 male ketogenic: 15.5 ± 7.1%, *P* = 0.001, CI = 5.8–18.0%; Fig. 1 and Supplementary Table 3). Conversely, B6 females and FVB males and females fed the same two diets did not show diet-dependent differences of body fat percentage (B6 female American: 20.7 ± 8.8%, B6 female ketogenic: 17.2 ± 5.7%; FVB male American: 18.1 ± 6.9%, FVB male ketogenic: 20.4 ± 7.5%; FVB female American: 13.4 ± 6.7%, FVB female ketogenic: 16.2 ± 6.4%; Fig. 1 and Supplementary Table 3). The mean fat percentage in B6 males on the ketogenic diet is lower than the fat percentage in FVB males on the ketogenic diet, although the difference is not statistically significant (Fig. 1 and Supplementary Table 3).

### Sex modulates hybrid population fat differences

The F1 males, but not females, also responded to the ketogenic diet. F1 males on the American diet had 1.25-fold higher body fat percentage than on the ketogenic diet (F1 male American: 32.5 ± 3.2%, F1 male ketogenic: 25.9 ± 4.7%, *P* = 0.020, CI = 1.3–11.9%; Fig. 1 and Supplementary Table 3). We also observed that the F1 males on the ketogenic diet had a 1.67-fold higher fat percentage than that observed in B6 males on the ketogenic diet (F1 male ketogenic: 25.9 ± 4.7%, B6 male ketogenic: 15.5 ± 7.1% *P* = 0.005, CI = 3.7–15.6%; Fig. 1 and Supplementary Table 3).

### Sex and diet modulate phenotypes in the F2 population

As expected, sex had a profound effect on the phenotypes used for analysis (Supplementary Table 3). This effect was greater than the effect of diet for all phenotypes (Supplementary Table 3). Regardless of the carbohydrate composition of the diet, females had lower fat mass gain than males (F2 females, 4.5 ± 3.0 g, F2 males, 8.2 ± 4.5 g, *P* < 0.001, CI = 2.8–4.2 g), lower lean mass gain than males (F2 females, 5.5 ± 1.6 g, F2 males, 9.0 ± 3.2 g, *P* < 0.001, CI = 3.0–3.9 g), and lower serum HDL cholesterol concentration than males (F2 females, 157.6 ± 58.3 ng/mL, F2 males, 201.6 ± 32.5 ng/mL, *P* < 0.001, CI = 34.9–53.0 ng/mL).

Diet had a significant effect on lean mass gain and serum HDL cholesterol (Supplementary Table 3). Irrespective to sex, F2s on the ketogenic diet gained less lean mass than F2s on the American diet (F2 ketogenic, 6.9 ± 2.8 ng/mL, F2 American, 7.4 ± 3.2 ng/mL, *P* = 0.002, CI = 0.3–1.2 ng/mL) and had lower serum HDL cholesterol concentration on the ketogenic diet compared to F2s on the American diet (F2 ketogenic, 175.1 ± 52.2 ng/mL, F2 American, 183.3 ± 52.4 ng/mL, *P* = 0.027, CI = 1.2–19.2 ng/mL).

A significant interaction of sex and diet (Supplementary Table 3) was observed for lean mass gain where F2 males on the ketogenic diet gain significantly less lean mass than F2 males on the American diet (F2 male ketogenic, 8.2 ± 3.1 g, F2 male American, 10.0 ± 3.0 g, *P* < 0.001, CI = 0.9–2.7 g).

The amount of fat mass gain was highly heritable in the F2 population. Broad-sense heritability for the trait was 81% in males and 71% in females.

### Linkage analysis reveals QTLs for a fat mass gain during the feeding trial

**Fig. 2:**
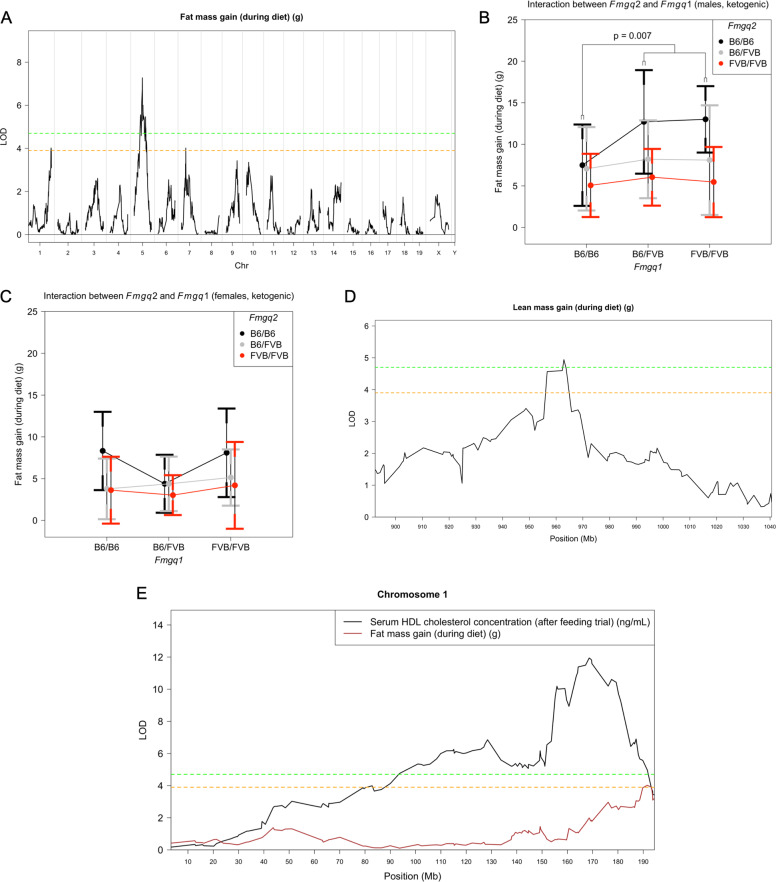
QTL profiles and interaction plots. The QTL profiles show the logarithmic *P* values across the whole genome. Positive values indicate that the FVB allele increases the trait, while negative values indicate that the FVB allele decreases the trait. The horizontal lines present the genome-wide thresholds of high significance (*P* < 0.01, green) and significance (*P* < 0.05, orange) based on 10,000 permutations of the data. **A** QTL profile for fat mass gain during the feeding trial. In the combined model including both sexes and both diets (y~ sex * diet + marker), a highly significant QTL is located on Chr5 at 73.7 Mb (*Fmgq2*) (black confidence interval). There are additional significant QTLs on Chr1 at 191.6 Mb (*Fmgq1*) and on Chr7 at 40.5 Mb (*Fmgq3*). The top marker at *Fmgq2* explains the most variation in fat mass gain in males on the ketogenic diet. **B** Interaction plots for *Fmgq2* and *Fmgq1* in ketogenic males and **C** ketogenic females represented as mean ± standard deviation. In ketogenic males, if *Fmgq2* is homozygous for the B6 allele and *Fmgq1* harbors at least one FVB allele, a functional interaction effect was evident. As long as *Fmgq2* carries one B6 and one FVB allele, the effects of the two loci are additive, independent of the genotype at *Fmgq1*. **D** QTL profile on Chr5 for lean mass gain during the 3-month feeding trial. In the combined model including both sexes and both diets (y~ sex * diet + marker), a highly significant QTL is located on Chr5 at 73.7 Mb (*Lmgq1*) (black confidence interval). The top marker at *Lmgq1* explains the most variation in a lean mass gain in males on the ketogenic. **E** QTL profile on Chr1 for serum HDL cholesterol concentration after the 3-month feeding trial. In the combined model that includes both sexes and diets (y~ sex * diet + marker), the peak position of the highly significant QTL is located at 168.6 Mb (*Hdlq1*) (black confidence interval).

In the combined analysis that incorporates all F2s on both diets (y~ sex * diet + marker), we detected QTL for fat mass gain on Chr1 at 191.6 Mb (*Fmgq1*; *P* < 0.001, CI = 180.2–194.4 Mb), Chr5 at 73.7 Mb (*Fmgq2*; *P* < 0.001, CI = 66.1–76.6 Mb), and Chr7 at 40.5 Mb (*Fmgq3*; *P* < 0.01, CI = 36.6–44.5 Mb) (Fig. 2A and Table 1). At *Fmgq1*, the FVB allele contributes to higher fat mass gain, and the top marker (UNC010475128) accounts for 3.44% of the variance. *Fmgq2* is a highly significant QTL where the FVB allele contributes to lower fat mass gain. The top marker (backupUNC050383757) accounts for 6.3% of the total variance in the F2 population (Table 1). The top marker explains 22.8% of the variation in males on the ketogenic diet, while it only explaining 5.9% of the variation in females on the ketogenic diet and 4.1% and 1.6% of the variation in males and females on the American diet, respectively. At *Fmgq3*, the FVB allele contributes to lower fat mass gain, and the top marker (JAX00150446) accounts for 3.4% of the variance in fat mass gain.

**Table 1:** Significant QTLs for fat mass and lean mass gain during feeding trial and for serum HDL cholesterol concentration after feeding trial.

Phenotype	Model	Chr	Left marker (Mb)	Top marker (Mb)	Right marker (Mb)	LOD (−log10(*P* value))^ξ^	Effect of B6/FVB alleles^ξ^	Effect of FVB/FVB alleles^ξ^	Variance explained (%)^ξ^
Fat mass gain (during feeding trial) (g)	Combined (both sexes, both diets) (y ~ sex * diet + marker)	1	180.2	191.6	194.4	4.02^b^	1.44	2.32	3.44
	Combined (both sexes, both diets) (y ~ sex * diet + marker)	5	66.1	73.7	76.6	7.28^a^	−1.95	−2.78	6.29
	Combined (both sexes, both diets) (y ~ sex * diet + marker)	7	36.6	40.5	44.5	4.02^b^	0.23	−1.70	3.43
Lean mass gain (during feeding trial) (g)	Combined (both sexes, both diets) (y ~ sex * diet + marker)	5	66.1	73.7	76.6	4.94^a^	−0.78	−1.62	3.34
Serum HDL concentration (after feeding trial) (ng/mL)	Combined (both sexes, both diets) (y ~ sex * diet + marker)	1	160.6	168.6	176.1	11.94^a^	26.88	48.01	9.93

^ξ^LOD scores, allele effects, and variance explained are provided for the top marker in each confidence interval. Effects of the B6/FVB and FVB/FVB alleles are given relative to B6/B6 alleles at this location.

^a^Genome-wide significance threshold of high significance (*P* < 0.01) based on 10,000 permutations.

^b^Genome-wide significance threshold of significance (*P* < 0.05) based on 10,000 permutations.

We expected that FVB alleles would drive higher fat mass gain across the genome based on the initial screen of B6 and FVB showing that B6 males have a differential response to American and ketogenic diets, while FVB mice do not have a differential response to these two high-fat diets. Surprisingly, the FVB allele contributes to lower fat mass gain in response to the ketogenic diet at the most highly significant QTL at *Fmgq2* as well as *Fmgq3*, but higher fat mass gain at *Fmgq1*.

In the initial analysis of fat percentage in B6, FVB, and F1 populations, we also observed that the male response to the ketogenic diet was greater in the F1 population compared to the B6 parent strain (Fig. 1). This prompted us to test if the combination of parent alleles at *Fmgq1* with either *Fmgq2* or *Fmgq3* resulted in a higher fat mass gain in the F2 population. We found an interaction between *Fmgq2* and *Fmgq1* (*P* = 0.007, CI = 1.58–9.07 g) affecting fat mass gain. The interaction effect was specific to males on the ketogenic diet. Males that are homozygous for the B6 allele at *Fmgq2* and that carry at least one FVB allele at *Fmgq1* gained about 5 g more fat on a ketogenic diet than males homozygous for the B6 allele at both loci (Fig. 2B, C).

### Linkage analysis reveals QTLs for a lean mass gain during the feeding trial

In the combined model that includes male and female F2s on both diets (y~ sex * diet + marker), we detected a significant QTL for lean body mass gain during the 3-month feeding trial (*Lmgq1;* *P* < 0.001, CI = 66.1–76.6 Mb) (Table 1 and Fig. 2D). *Lmgq1* overlapped with *Fmgq2*. We observe again that males on the ketogenic diet drive this QTL. In the combined model, the top marker (backupUNC050383757) explains 3.3% of the variance in lean mass gain. In males on the ketogenic diet, the top marker (JAX00131700) explains 19.0% of the variation in lean mass gain while it only explaining 1.2% of the variation in females on the ketogenic diet and 8.2% and 4.1% of the variation in males and females on the American diet, respectively.

### Linkage analysis reveals QTLs for serum HDL concentration at the end of the feeding trial

**Fig. 3:**
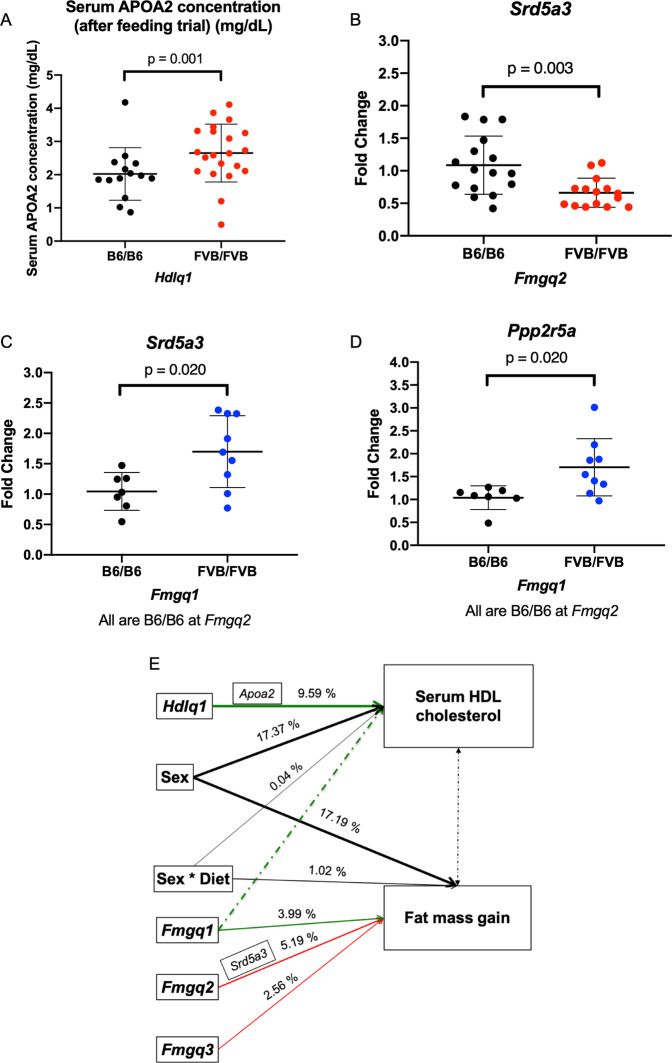
Candidate genes and models. **A** Serum *APOA2* concentration for F2 animals which are homozygous for the alternative alleles at *Hdlq1*. The FVB/FVB genotype at *Hdlq1* drives higher serum *APOA2* concentration in addition to higher serum HDL cholesterol concentration (B6/B6 *n* = 14, FVB/FVB *n* = 24, Wilcoxon two-sample *t* test). **B** Fold change in *Srd5a3* expression among F2 animals of genotype FVB/FVB (*n* = 15) relative to B6/B6 (*n* = 16) at *Fmgq2*. A decrease in *Srd5a3* expression is associated with the FVB/FVB genotype at Fmgq2 relative to the B6/B6 genotype at *Fmgq2* (Welch’s two-sample *t* test when variances are unequal). **C** Fold change in *Srd5a3* expression among F2 animals of different genotypes at *Fgmq1* and *Fmgq2*. A 1.59-fold increase in *Srd5a3* expression is associated with F2 animals that are homozygous for the FVB allele at *Fmgq1* and B6/B6 at *Fmgq2* (*n* = 9) relative to those that are homozygous for the B6 allele at both loci (*n* = 7) (Welch’s two-sample *t* test when variances are unequal). **D** Fold change in *Ppp2r5a* expression among F2 animals of different genotypes at *Fgmq1* and *Fmgq2* (Welch’s two-sample *t* test when variances are unequal). A 1.61-fold increase in *Ppp2r5a* expression is associated with F2 animals that are homozygous for the FVB allele at *Fmgq1* and B6/B6 at *Fmgq2* (*n* = 9) relative to those that are homozygous for the B6 allele at both loci (*n* = 7) (Wilcoxon two-sample *t* test). **E** Graphical representation of SEM for fat mass gain during the feeding trial and serum HDL cholesterol concentrations after the feeding trial in the combined model that contains both sexes and both diets. Solid arrows indicate the direction of paths and the weight of each arrow is proportional to the path coefficient from the predictor to the variable and the percentage of variation in the variable that is explained by each predictor. Positive effects (green arrows) indicate that the FVB allele increases the trait; negative effects (red arrows) indicate that the FVB allele decreases the trait. The single-headed dashed arrow represents the inferred pleiotropic causal pathway at *Fmgq1* that was identified in the causal network inference for fat mass gain and serum HDL cholesterol concentration. The double-headed dashed arrow represents a covariate pathway detected by the structural model between fat mass gain and serum HDL cholesterol concentration.

In the combined model that includes male and female F2 animals on both diets (y~ sex * diet + marker), we detected a highly significant QTL on Chr1 at 168.6 Mb (*Hdlq1*) for serum HDL concentration after the 3-month feeding trial (*P* < 0.001, CI = 160.6–176.1 Mb; Fig. 2E). All genotype classes (B6/B6 155.4 ± 48.8 ng/mL; B6/FVB 179.2 ± 51.5 ng/mL; FVB/FVB 200.6 ± 48.5 ng/mL) are significantly different from each other at this locus (B6/B6: B6/FVB *P* < 0.001, CI = 9.7–38.1 ng/mL; B6J/B6J: FVB/FVB *P* < 0.001, CI = 28.7–61.8 ng/mL; FVB/FVB: B6/FVB *P* < 0.001, CI = 7.4–35.4 ng/mL; Supplementary Fig. S5). This locus harbors the *Apoa2* gene which has previously been associated with serum HDL cholesterol concentration [12–14]. Therefore, we measured serum APOA2 concentrations in F2s with the highest and lowest serum HDL cholesterol concentrations. These measurements confirm significant differences between homozygous genotype classes at *Hdlq1* (B6/B6 2.2 ± 0.8 mg/dL; FVB/FVB 3.0 ± 0.9 mg/dL, *P* = 0.004, CI = 0.3–2.2 mg/dL; Fig. 3A).

### KEGG pathway annotation of candidate genes at *Fmgq1* and *Fmgq2* highlights steroid hormone biosynthesis

We searched for candidate genes that might elucidate the functional interaction between *Fmgq1* and *Fmgq2*. Out of 98 positional candidate genes, 4 genes at *Fmgq1* are found on metabolically relevant KEGG pathways: *Hsd11b1* (steroid hormone biosynthesis; mmu00140), *Ephx1* (bile secretion; mmu004976), *Camk1g* (aldosterone synthesis and secretion; mmu004925), and *Ppp2r5a* (AMPK signaling pathway; mmu004152) (Table 2). Out of 482 positional candidate genes, 2 genes at *Fmgq2* are found on metabolically relevant KEGG pathways: *Srd5a3* (steroid hormone biosynthesis; mmu00140) and *Cox7b2* (nonalcoholic fatty liver disease; mmu004932) (Table 2).

**Table 2:** Candidate genes in QTL confidence intervals for *Fmg1* and *Fmg2* were annotated with metabolically relevant KEGG pathways.

Phenotype	Model	QTL	KEGG pathway	Gene symbol (MGI)
Fat mass gain (during feeding trial) (g)	Combined (both sexes, both diets) (y ~ sex * diet + marker)	*Fmgq1*	Steroid hormone biosynthesis (mmu00140)	*Hsd11b1*
			Bile secretion (mmu04976)	*Ephx1*
			Aldosterone synthesis and secretion (mmu04925)	*Camk1g*
			AMPK signaling pathway (mmu04152)	*Ppp2r5a*
	Combined (both sexes, both diets) (y ~ sex * diet + marker)	*Fmgq2*	Steroid hormone biosynthesis (mmu00140)	*Srd5a3*
			Nonalcoholic fatty liver disease (mmu04932)	*Cox7b2*

On the steroid hormone biosynthesis pathway, *Srd5a3* converts inactive testosterone to active dihydrotestosterone. This makes *Srd5a3* a particularly interesting candidate at *Fmgq2* given the sex specificity we see at this QTL and the functional interaction we observed between *Fmgq2* and *Fmgq1*. Consistent with *Srd5a3* underlying *Fmgq2*, we observed lower expression of *Srd5a3* in gonadal fat of F2s that are homozygous for the FVB allele at *Fmgq2* relative to F2s that are homozygous for the B6 allele at *Fmgq2* (Fig. 3B). Interestingly, we also observe a 1.59-fold increase in *Srd5a3* expression in F2s that are B6/B6 at *Fmgq2* and carry FVB/FVB alleles at *Fmgq1* relative to F2s that are B6/B6 at both loci (Fig. 3C). This makes *Srd5a3* a strong candidate gene at *Fmgq2* and for the functional interaction between *Fmgq1* and *Fmgq2*.

At *Fmgq1*, we did not observe any genotype-dependent differences in expression of *Hsd11b1* (liver)*, Ephx1* (liver), or *Ppp2r5a* (gonadal fat) (Supplementary Fig. S6ABC). We did, however, observe a 1.61-fold increase in *Ppp2r5a* expression in F2s that are B6/B6 at *Fmgq2* and carry FVB/FVB alleles at *Fmgq1* relative to F2s that are B6/B6 at both loci (Fig. 3D). This makes *Ppp2r5a* a strong candidate gene at *Fmgq1* for the functional interaction between *Fmgq1* and *Fmgq2*.

The remaining candidate at *Fmgq1, Camk1g*, is exclusively expressed in brain tissue, which was unavailable to measure expression. Given the involvement of *Camk1g* in aldosterone synthesis and secretion, we instead measured serum aldosterone concentration (after the feeding trial) and observed no genotype-dependent differences (Supplementary Fig. S6D). The remaining candidate at *Fmgq2*, *Cox7b2* is expressed exclusively in the testis, which was unavailable to measure its expression.

### Conditioned linkage analysis of fat and lean mass gain

*Fmgq2* and *Lmgq1* overlap in the combined model of F2s that includes both sexes and diets. For both traits, this QTL appears to be driven largely by males on the ketogenic diet. This shared QTL prompted us to model the relationship between fat and lean mass gain. There is no significant change to the LOD score on Chr5 at 73.7 Mb when the fat mass gain is conditioned on the lean mass gain, nor when the lean mass gain is conditioned on fat mass gain. Thus, the relationship between fat and lean mass gain at this locus could not be elucidated in this model. This would suggest that the overlapping QTL region harbors one or two tightly linked genes that affect both lean and fat mass in the same direction.

### Conditioned linkage analysis of fat mass gain and serum HDL cholesterol concentration

The close proximity of *Hdlq1* and *Fmgq1* in the combined models prompted us to model the relationship between fat mass gain and serum HDL cholesterol concentration at these loci. We observed that when the fat mass gain is conditioned on serum HDL cholesterol concentration, there is no significant change to the LOD score on Chr1 at 191.6 Mb (Table 3). Thus, the relationship between fat mass gain and serum HDL cholesterol concentration could not be elucidated in this model. Similarly, when serum HDL cholesterol concentration is conditioned on the fat mass gain, the LOD score on Chr1 at 168.6 Mb drops by only 1.08 LOD suggesting that *Hdlq1* is not shared between fat mass gain and serum HDL cholesterol concentration (Table 3).

**Table 3:** Structural equation model for fat mass gain during feeding trial and serum HDL cholesterol concentration after the feeding trial.

Conditioned and unconditioned QTL scans				
Phenotype	Model	Condition	QTL	LOD (−log10(*P* value))^ξ^
Fat mass gain (during feeding trial) (g)	Combined (both sexes, both diets) (y ~ sex * diet + marker)	Unconditioned	*Fmgq1*	4.02*
	Combined (both sexes, both diets) (y ~ sex * diet + m + HDL)	Serum HDL cholesterol concentration (after feeding trial) (ng/mL)	*Fmgq1*	4.58*
Serum HDL concentration (after feeding trial) (ng/mL)	Combined (both sexes, both diets) (y ~ sex * diet + marker)	Unconditioned	*Hdlq1*	11.94
	Combined (both sexes, both diets) (y ~ sex * diet + marker + fat mass gain)	Fat mass gain (during feeding trial) (g)	*Hdlq1*	10.86
*Causal network analysis*				
Relationship	Model	*P* value	AIC	BIC
Fat mass gain ← serum HDL cholesterol	(Fat mass gain ~ HDL) ~ sex * diet + top marker at *Fmgq1*	0.0046^a^	2181	2209
Fat mass gain → serum HDL cholesterol	(HDL ~ fat mass gain) ~ sex * diet + top marker at *Fmgq1*	0.0252^a^	4204	4232
*Structural model*				
	Variable	Predictor	Path coefficient (% variation explained)	t-statistic of path coefficient
	Fat mass gain (during feeding trial) (g)	Sex	0.43 (17.19%)	9.89
		Sex * diet	−0.09 (1.02%)	−2.03
		*Fmgq1*	0.19 (3.99%)	4.40
		*Fmgq2*	−0.21 (5.19%)	−4.87
		*Fmgq3*	−0.11 (2.56%)	−2.55
	Serum HDL cholesterol concentration (after feeding trial) (ng/mL)	Sex	0.44 (17.37%)	10.09
		Sex * diet	−0.01 (0.04%)	−2.03
		*Hdlq1*	0.31 (9.59%)	7.20

^ξ^LOD (−log10(*P* values)) is provided for the top marker in each confidence interval.

^a^Causal inference is significant at Holm–Bonferroni corrected *P* < 0.05.

### Causal network between *Fmgq1*, fat mass gain, and serum HDL cholesterol concentration

We also explored causal networks between fat mass gain and serum HDL cholesterol concentration at *Fmgq1*. After adjusting for multiple testing, the inferred network shows that serum HDL cholesterol concentration and fat mass gain are independently related to *Fmgq1* (Table 3). The AIC and BIC model selection scores suggest that the model in which serum HDL cholesterol concentration occurs upstream of fat mass gain at *Fmgq1* is most consistent with the data (Table 3).

### Structural equation modeling of fat mass gain and serum HDL cholesterol concentration

We built a structural model from this inferred network to illustrate the magnitude of the effects of each predictor in the models of fat mass gain and serum HDL cholesterol concentration (Fig. 3E). The path coefficients are all significantly different from zero (Table 3). The proportion of variation explained with the predictors modeled for fat mass gain and serum HDL cholesterol concentration is 29.95% and 27.00%, respectively.

## Discussion

This study provides evidence that individual responses to a high-fat diet significantly depends upon three factors: (1) the presence or absence of carbohydrates in the high-fat diet; (2) the combination of alleles occurring in the study population; and (3) sex. In our experiment, B6 males have a lower percentage of body fat in response to the high fat, no carbohydrate, ketogenic diet, while in contrast, B6 females and FVB males and females do not have a differential response to American and ketogenic diets. This sex-specific response to carbohydrate restriction on ketogenic diet observed in B6 males persists in F1s males, and we observed that the combination of B6 and FVB alleles in the hybrids results in a higher percentage of body fat in response to the feeding trial. The difference in ages at the beginning of the parent and F1 feeding trials might also contribute to the higher percentage of body fat we observed in the F1s. In the F2 population, we observed a very high heritability for fat mass gain in both sexes and were able to identify significant QTLs for fat mass gain at *Fmgq1*, *Fmgq2*, and *Fmgq3*. This raises the question of whether these loci could be used to make predictions about the individual responses to carbohydrate restriction. *Fmgq2* explains the most variation in the fat mass gain in males on the ketogenic diet. We observed that the FVB allele drives lower fat mass gain at *Fmgq2* and *Fmgq3*, while at *Fmgq1* the FVB allele drives higher fat mass gain. In addition, we provided evidence that male F2s exposed to the ketogenic diet that is homozygous for B6 alleles at *Fmgq2* and carry at least one FVB allele at *Fmgq1* gain the most fat mass during the feeding trial. We observed that *Fmgq2* and *Lmgq1* represent the same QTL on Chr5 for fat and lean mass gain. Likely, a single gene or two tightly linked genes are responsible for the change in lean and fat mass.

The QTL for serum HDL cholesterol concentration at *Hdlq1* is significant in both males and females and contains *Apoa2. Apoa2* has been described repeatedly for its relationship to serum HDL cholesterol concentration, especially in these two strains [12–14]. The initial observation of the *Apoa2* locus affecting serum concentrations of HDL cholesterol was made in an association study of females using an advanced intercross line between B6 and NZB/BINJ [12]. Later, in a panel of inbred strains that included B6 and FVB mice, it was determined that the amino acid substitution Ala61Val in APOA2 led to increased serum HDL concentrations [13]. B6 carry Ala61 while FVB mice carry amino acid substitution 61Val. Both males and females of all strains carrying the 61Val had increased serum HDL cholesterol concentrations. The results of our linkage analysis for serum cholesterol concentrations are consistent with the amino acid differences. They show that *Hdlq1* affects HDL in males and females. We confirmed that FVB alleles at *Hdlq1* drive higher concentrations of serum HDL cholesterol and serum APOA2.

In our causal network of the relationship between fat mass gain and serum HDL cholesterol concentration, we found that serum HDL cholesterol concentration and fat mass gain are independently linked to *Fmgq1*. The close proximity of the *Fmgq1* and *Hdlq1* QTLs puts them in linkage disequilibrium and this makes their direct and indirect effects difficult to detangle. In general, we would have expected that serum HDL cholesterol concentration would have a negative relationship with fat mass gain given the well-established relationship between serum HDL cholesterol and obesity in humans. It is likely that the two traits are independently linked to *Fmgq1* because of the linkage disequilibrium. However, the AIC and BIC scores for the models suggest that differences in serum HDL cholesterol concentration occur upstream of the differences we observe in fat mass gain at *Fmgq1*.

Overlaying genes within *Fmgq1* and *Fmgq2* onto metabolically relevant KEGG pathways revealed candidate genes involved in the biogenesis of cortisol (*Hsd11b1*), aldosterone (*Camk1g*), and testosterone (*Srd5a3*). Cholesterol falls upstream of the production of each of these steroid hormones. *Ephx1* resides inside of *Fmgq1 and* plays a role in the transfer of bile acids to the liver, a process that is critical for the endogenous synthesis of cholesterol. Each of these candidate genes has in common, a relationship with cholesterol. This offers insight into the role these particular genes could play in the causal network we inferred that showed serum HDL cholesterol concentration occurring upstream of fat mass gain. The inferred network might be reflective of the relationship between fat mass gain and one of these candidate genes more so than a direct relationship between serum HDL cholesterol concentration and fat mass gain.

Further, our candidate genes offer insight into the sex-specific interaction in males on the ketogenic diet that we observed between *Fmgq1* and *Fmgq2*. Our most prominent QTL at *Fmgq2* harbors *Srd5a3* that converts inactive testosterone to active dihydrotestosterone. We confirmed that F2s that are homozygous for FVB alleles at *Fmgq2* express *Srd5a3* less than F2s that are homozygous for B6 alleles. At this QTL, FVB alleles also drive lower fat mass gain. This suggests that more active dihydrotestosterone promotes more fat mass gain at *Fmgq2*. The functional interaction we observed between *Fmgq1* and *Fmgq2* showed us that male F2s that are homozygous B6 at *Fmgq2* and carry at least one FVB allele at *Fmgq1* gained the most fat mass. Interestingly, we observed that F2s that carry this interaction have a 1.59-fold increase in expression of *Srd5a3* relative to animals that are homozygous B6 at both *Fmgq1* and *Fmgq2*. This provides further evidence that more active dihydrotestosterone promotes more fat mass gain in this population. *Ppp2r5a* at *Fmgq1* has been associated with polycystic ovary syndrome (PCOS) through pathway analysis of genes enriched in genome-wide association studies (GWAS). *Ppp2r5a* is a member of the oocyte meiosis KEGG pathway that was significantly associated with PCOS, a pathway affected by the hyperandrogenemia that frequently occurs with PCOS [15]. The association *of Ppp2r5a* with PCOS makes *Srd5a3*, and *Ppp2r5a* primary candidate genes of interest for further investigation.

Each of the remaining candidate genes has a potential relationship with *Srd5a3* and active dihydrotestosterone levels. *Hsd11b1* at *Fmgq1* reversibly converts active cortisol to inactive cortisone. Epidemiological data in humans show that the ratio of androgens to glucocorticoids is critical to metabolic homeostasis and alterations in this ratio can lead to increased incidence of obesity and metabolic syndrome [16]. In addition, excessive levels of active dihydrotestosterone have been shown to stimulate aldosterone secretion by a mechanism that is dependent upon the calmodulin/calmodulin-dependent protein kinase and protein kinase C intracellular signaling pathways [17]. *Camk1g* at *Fmgq1* is a member of this pathway and people with obesity often experience hyperaldosteronism [18]. *Ephx1* at *Fmgq1* has previously been associated with other *Srd5a* isoforms in GWAS of PCOS [19], and similarly, *Cox7b2* at *Fmgq2*, has been identified in another GWAS for PCOS [20]. The association with PCOS establishes a probable link between both *Ephx1* and *Cox7b2* and abnormal androgen secretion.

Sequence variation between B6 and FVB result in amino acid changes in EPHX1, CAMK1G, and PPP2R5A, sometimes at multiple locations within the proteins. In HSD11B1 and SRD5A3, no amino acid changes have been documented, but sequence variation in *Hsd11b1* and *Srd5a3* does result in noncoding transcript variants that might be of interest in further investigation. These noncoding transcript variants would suggest differences in expression of relevant candidate genes like we observed for *Srd5a3*. However, we did not observe genotype-dependent differences in expression of *Hsd11b1*, but did find in the F2 population that mice that are B6/B6 at *Fmgq2* and carry FVB/FVB alleles at *Fmgq1* express *Ppp2r5a* 1.61-fold more than F2s that are B6/B6 at both loci.

Overall, our data parallel a clinical trial showing a greater fat mass loss among men than women on very low energy, ketogenic diet [21–23]. The differences could not be attributed to the attenuation of appetite-stimulating hormones, and concentrations of serum steroid hormones were not considered in the study. Unfortunately, studies in humans that utilize very low carbohydrate, ketogenic diets typically lack the power to detect sex differences or fail to incorporate realistic control diets [24–28]. Nonetheless, meta-analyses of human, genome-wide association studies of body mass index and waist-to-hip circumference have identified several sex-specific loci [29–33]. Future sex-specific genome-wide association studies that are powered to detect differential responses to macronutrients between men and women are needed.

## Conclusions

It is possible that the genetic architecture for fat gain in response to high-fat diets is more complex in females than in males, and as such, the genetic component we are able to detect in our model contributes more to the overall response in males than in females. The observation of these sex-specific QTLs for fat and lean mass gain at *Fmgq2* and *Lmgq1* and serum HDL cholesterol concentration at *Hdlq2* and *Hdlq3* highlights the importance of sex-specific analyses during the development of individualized dietary guidelines. We identified a known QTL at *Hdlq1* for serum HDL cholesterol concentration that harbors *Apoa2*. Interestingly, the QTLs at *Fmgq2* and *Fmgq3* affect fat gain directly while *Fmgq1* seems to influence fat gain directly as well as via an intermediate physiological change in serum cholesterol related to the strain-specific *Apoa2* phenotypes. We have shown that genotypes at *Fmgq2* alter the expression of *Srd5a3* in a sex- and genotype-specific manner. It remains unclear if these loci explain the differential response to carbohydrate restriction we observed in the initial parent strain screen or if instead, they characterize the increased fat mass gain that we observe after the parental genomes are combined in hybrid and F2 populations. Further investigation of the candidate genes presented here will elucidate their roles in response to carbohydrate restriction in the parent, hybrid, and F2 populations.

## Supplementary information

Supplementary Figures

Supplementary Tables
