# Supplementary material for: Sex-specific genetic architecture in response to American and ketogenic diets
Source: Int J Obes (Lond). 2021 Mar 15;45(6):1284–97. doi: 10.1038/s41366-021-00785-7 (PMC8159743; doi:10.1038/s41366-021-00785-7)
Supplement: Supplementary file 1 — Supplementary Figures [file 41366_2021_785_MOESM1_ESM.pdf]

Supplementary Figures

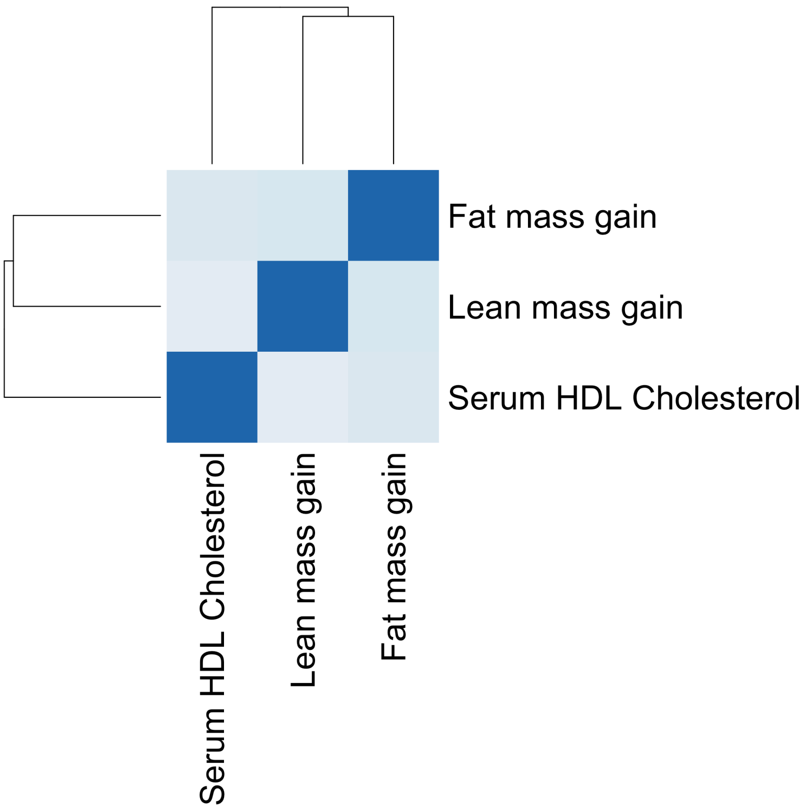

Figure S1. Plot depicting the correlation between examined phenotypes. Range of correlation coefficients: blue: r = 1, red: r = -1.

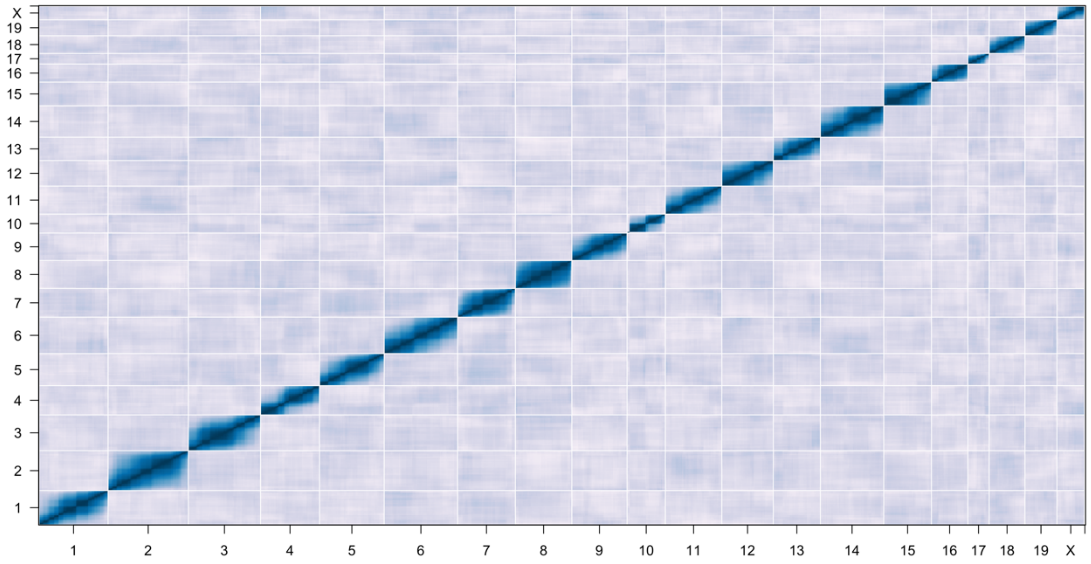

Figure S2. Genetic map of 16667 informative SNPs after phasing to the founder lines.

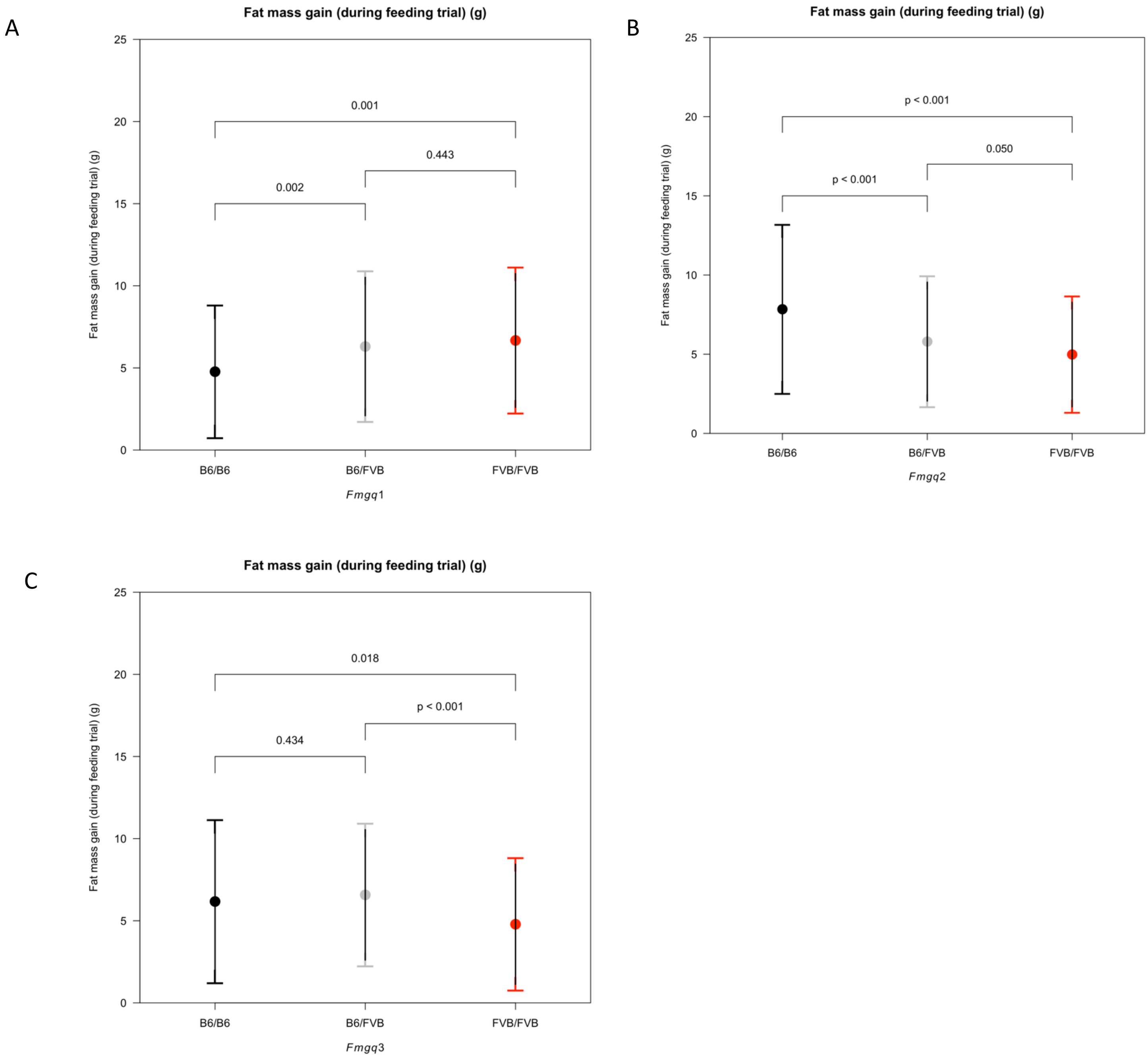

Figure S3. Fat mass gain effect plots. **A)** *Fmgq1,* combined model. **B)** *Fmgq2,* combined model. **C)** *Fmgq3,* combined model.

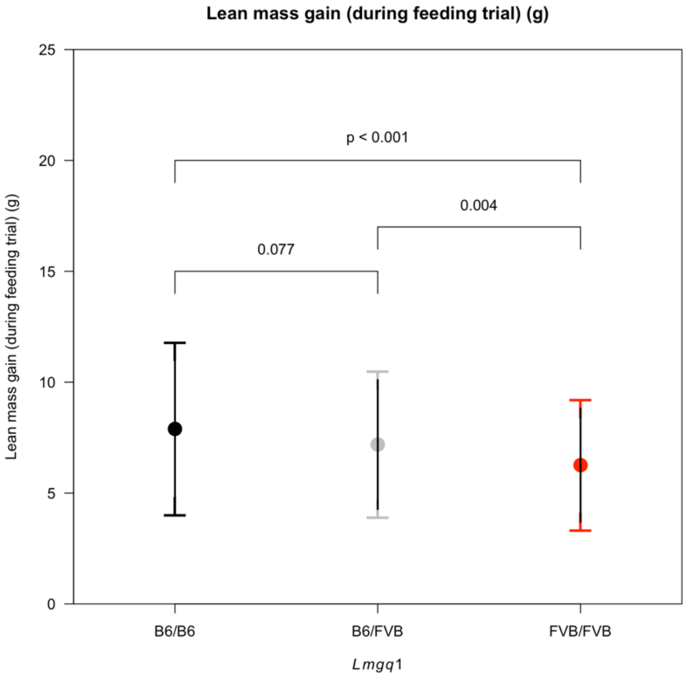

Figure S4. Lean mass gain effect plot for *Lmgq1* in the combined model.

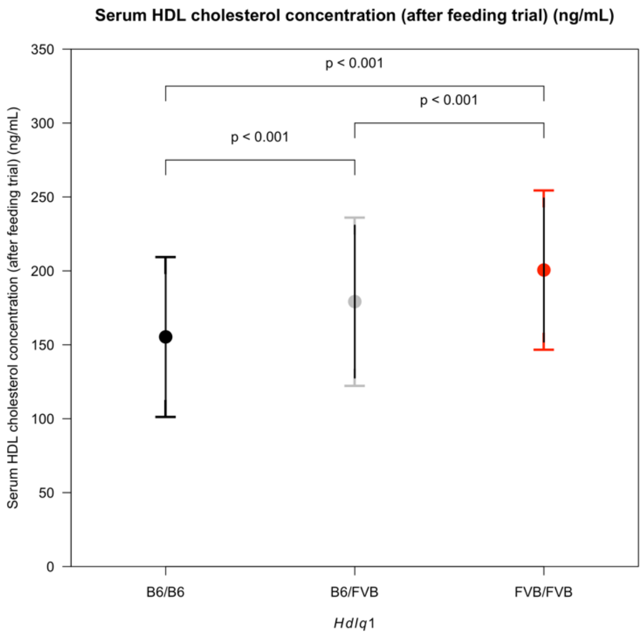

Figure S5. Serum HDL cholesterol concentration effect plots for *Hdlq1* in the combined model.

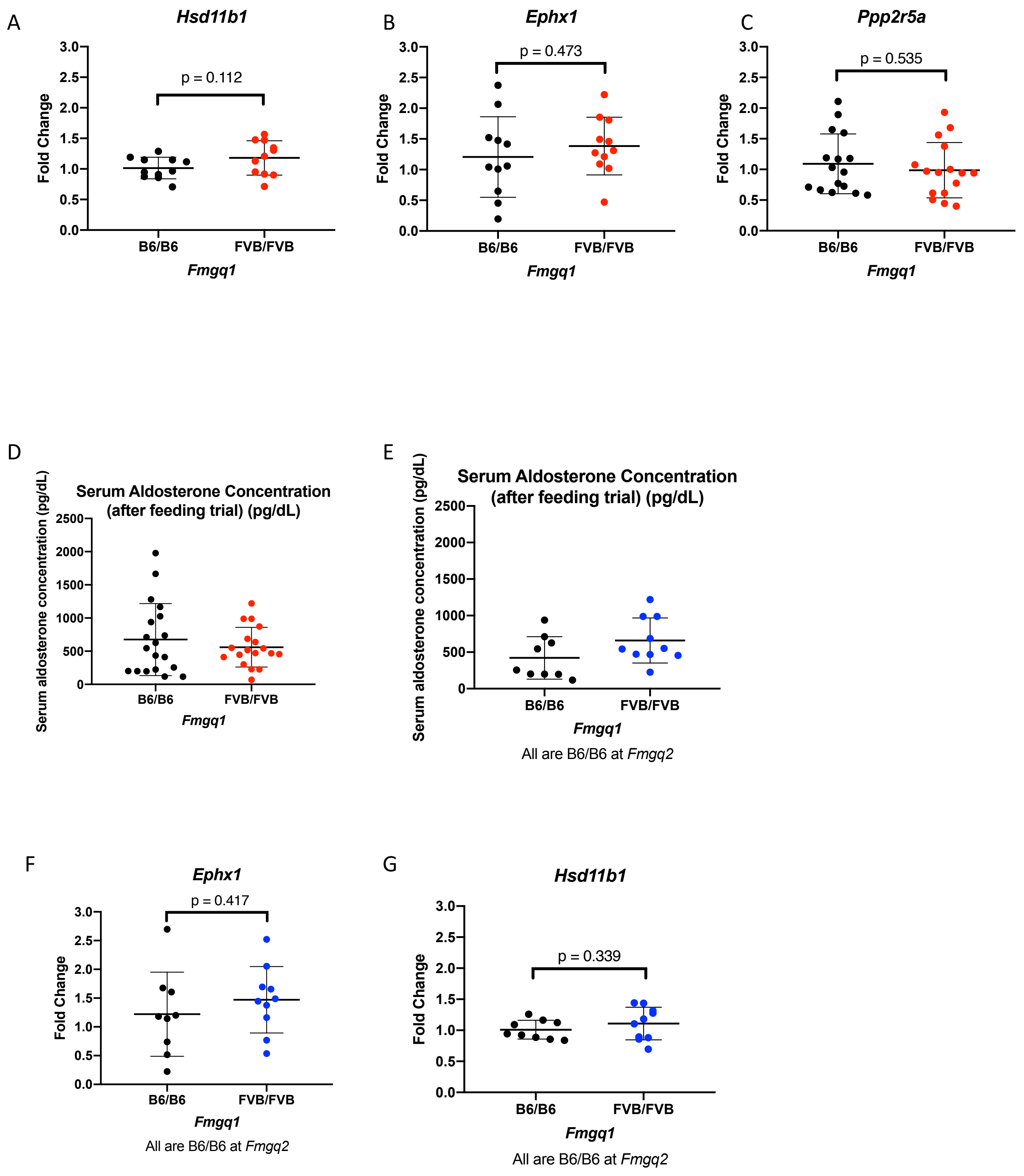

Figure S5. Candidate gene expression association with top QTL markers.
