# Supplementary material for: Sex-specific genetic architecture in response to American and ketogenic diets
Source: Int J Obes (Lond). 2021 Mar 15;45(6):1284–97. doi: 10.1038/s41366-021-00785-7 (PMC8159743; doi:10.1038/s41366-021-00785-7)
Supplement: Supplementary file 2 — Supplementary Tables [file 41366_2021_785_MOESM2_ESM.pdf]

Supplementary Tables

Supplementary Table S1. Diet compositions

Ingredient	D12052705C (American) (g)	D12052706 (Ketogenic) (g)
Casein	38.5	160
Soy Protein Isolate, Supro 661	0	0
Fish Protein Isolate	8.5	0
Egg White	55	0
Beef, Cooked	76.9	0
L-Cystine	3	3
Corn Starch	**30**	0
Wheat Starch	195	0
Potato Starch	**30**	0
Sucrose	205	0
Fructose	22	0
Cellulose, BW200	18.2	37.5
Inulin	**6**	12.5
Corn Oil	34.4	8
Menhaden Oil (299 ppm tBHQ)	1	8
Butter, Anhydrous	54.1	161
Lard	0	161
Flaxseed Oil	1	0
Olive Oil	27.5	0
t-BHQ	0.0047	0.0033
Mineral Mix S10026	10	10
Dicalcium Phosphate	13	13

Calcium Carbonate	5.5	5.5
Potassium Citrate, 1 H_2O	16.5	16.5
Vitamin Mix V10001	10	10
Biotin (1%)	0.1	0
Choline Bitartrate	2	2
Cholesterol	1.5	1.5
FD&C Red Dye #5	0	0.025
FD&C Blue Dye #1	0.05	0.025
Total	864.7547	609.5533
(g)		
Protein	140.7	142.2
Carbohydrate	455.3	3.1
Fat	139.7	339.9
Cholesterol	1.81	2.09
Fiber	22.7	46.9
% of Total Weight (g)		
Protein	16.3	23.3
Carbohydrate	52.6	0.5
Fat	16.2	55.8
Cholesterol	0.21	0.342
Fiber	2.6	7.7
Energy (kcal)		
Protein	563	569

Carbohydrate	1821	13
Fat	1257	3059
Total	3641	3641
% of Total Energy (kcal)		
Protein	15	16
Carbohydrate	50	0
Fat	35	84
% of total weight (g) Contribution to Protein (g)		
Casein	23.8	97.9
Fish Protein Isolate	5.7	0
Egg White	31.7	0
Beef, Cooked	36.6	0
L-Cystine	2.1	2.1
% of total weight (g) Contribution to Carbohydrate		
Corn Starch	5.9	0
Wheat Starch	38.4	0
Potato Starch	5.3	0
Sucrose	45.6	0
Fructose	4.9	0
Lipid Composition		
C2, Acetic	0	0
C4, Butyric	1.7	5.2
C6, Caproic	1	3.1
C8, Caprylic	0.6	1.8
C10, Capric	1.4	4.1

C12, Lauric	1.5	4.7
C14, Myristic	6.1	18.5
C14:1, Myristoleic	1	2.4
C15:0	0.1	0.2
C16, Palmitic	25.9	75.3
C16:1, Palmitoleic	2.4	6.7
C16:2	0	0.1
C16:3	0	0.1
C16:4	0	0.1
C17:0	0.2	0.6
C17:1, n-9	0.2	0
C18, Stearic	10.2	36.9
C18:1, Oleic, n-9	50.5	96.1
C18:1, Vaccenic, n-7	0.9	0
C18:2, Linoleic, n-6	26.5	47.9
C18:3, gamma-Linolenic, n-6	0	0
C18:3, alpha-Linolenic, n-3	2	4.6
C18:4, Stearidonic, n-3	0	0.2
C20, Arachidic	0.7	1.8
C20:1, n-9	0.2	1.1
C20:2, Eicosadienoic, n-6	0	1.3
C20:3, n-6	0	0.2
C20:3, n-3	0	0
C20:4, Arachidonic, n-6	0.1	0.6
C20:4, n-3	0	0
C20:5, Eicosapentaenoic, n-3	0.1	1.1
C21:0	0	0
C21:5, n-3	0	0.1
C22, Behenic	0	0
C22:1, Erucic	0	0

C22:4, Clupanodonic, n-6	0	0
C22:5, n-3	0	0.4
C22:5, n-6	0	0
C22:6, Docosahexaenoic, n-3	0.1	0.8
C24, Lignoceric	0	0
C24:1	0	0
Lipid Profile		
Saturated (g)	49.1	151.3
Monounsaturated (g)	54.1	106.4
Polyunsaturated (g)	29.0	57.3
Saturated (%)	36.7	47.9
Monounsaturated (%)	40.5	33.6
Polyunsaturated (%)	21.7	18.1
Total Omega-6 (g)	26.7	50.1
Total Omega-6 (%)	20.0	15.9
Total Omega-3 (g)	2.4	7.2
Total Omega-3 (%)	1.8	2.3
n6 : n3 ratio	11.3	6.9

Supplementary Table S2. qPCR primer sequences.

Gene	Forward Primer	Reverse Primer
B2m	GGTCTTTCTATATCCTGGCTCAC	ACATGTCTCGATCCCAGTAGA
Srd5a3	GGATGATAAGAATGTGTATGTTCTGG	TTTATACTGATGGGCGGATGAC
Hsd11b1	TTGGCCTCATAGACACAGAAAC	TGTGCCTTTGATGATCTCCAG
Ephx1	CTATGGCTTCAACTCCAGCTAC	GATGTCCAGCCCTTCAATCTT
Ppp2r5a	AAGTTTGTCCAACAGCTCCT	TGCTTTCTGATGAACGCTCT

Supplementary Table S3. Phenotypes in each study population.

Population	Phenotype	Sex (p-value)	Diet (p-value)	Sex * Diet (p-value)	Diet	Sex	Mean	+/-
B6	Fat percentage (after feeding trial) (% of body weight)	0.143	0.001	0.059	American	Male	27.4	5.2
						Female	20.7	8.8
					Ketogenic	Male	15.5	7.1
						Female	17.2	5.7
FVB	Fat percentage (after feeding trial) (% of body weight)	0.048	0.251	0.916	American	Male	18.1	6.9
						Female	13.4	6.7
					Ketogenic	Male	20.4	7.5
						Female	16.2	6.4
F1	Fat percentage (after feeding trial) (% of body weight)	< 0.001	0.007	0.088	American	Male	32.5	3.2
						Female	19.2	4.7
					Ketogenic	Male	25.9	4.7
						Female	19.3	4.7
F2	Fat mass gain (during feeding trial) (g)	< 0.001	0.373	0.120	American	Male	7.4	4.3
						Female	4.6	3.0
					Ketogenic	Male	8.5	4.6
						Female	4.4	2.9
	Lean mass gain (during feeding trial) (g)	< 0.001	0.003	< 0.001	American	Male	10.0	3.0
						Female	5.4	1.6
					Ketogenic	Male	8.2	3.1
						Female	5.6	1.7
	Serum HDL cholesterol (after feeding trial) (ng/mL)	< 0.001	0.027	0.956	American	Male	207.2	31.1
						Female	163.1	58.1
					Ketogenic	Male	197.3	33.0
						Female	152.6	58.3

Supplementary Table S4. Comprehensive list of all KEGG queries used for candidate gene associations.

Inclusion Criteria	KEGG id	KEGG pathway title
**Glucose and insulin related pathways**	mmu00010	Glycolysis/Gluconeogenesis
	mmu04910	Insulin signaling pathway
	mmu04931	Insulin resistance
**Diabetes Mellitus related pathways**	mmu04940	Type I diabetes mellitus
	mmu04930	Type II diabetes mellitus
	mmu04950	Maturity onset diabetes of the young (MODY)
**Fatty acid and adipocyte related pathways**	mmu00061	Fatty acid biosynthesis
	mmu04920	Adipocytokine signaling pathway
	mmu04923	Regulation of lipolysis in adipocytes
**Digestion and absorption related pathways**	mmu04973	Carbohydrate digestion and absorption
	mmu04974	Protein digestion and absorption
	mmu04975	Fat digestion and absorption
**Cholesterol related pathways**	mmu04979	Cholesterol metabolism
	mmu00140	Steroid hormone biosynthesis
	mmu00120	Primary bile acid biosynthesis
	mmu04927	Cortisol synthesis and secretion
	mmu04976	Bile secretion
	mmu04913	Ovarian steroidogenesis
	mmu00100	Steroid biosynthesis
	mmu04925	Aldosterone synthesis and secretion
	mmu04934	Cushing syndrome
	mmu03320	PPAR signaling pathway
	mmu04152	AMPK signaling pathway
**Additional Obesity and Metabolic Syndrome related pathways**	mmu04932	Non-alcoholic fatty liver disease (NAFLD)
	mmu04714	Thermogenesis
	mmu04371	Apelin signaling pathway
	mmu04146	Peroxisome
